# Correlation Between Non-Contrast Magnetic Resonance Imaging Findings and Clinical Assessment in Patients With Adhesive Capsulitis

**DOI:** 10.7759/cureus.79626

**Published:** 2025-02-25

**Authors:** Rodrigo A Beraldo, Thiago G Dotta, Henry F Moreira, Jorge Assunção, Eduardo Baptista, Mauro E Gracitelli, Eduardo A Malavolta

**Affiliations:** 1 Instituto de Ortopedia e Traumatologia, Hospital das Clínicas Faculdade de Medicina, Universidade de São Paulo, São Paulo, BRA; 2 Ortopedia e Traumatologia, Instituto Jundiaiense de Ortopedia e Traumatologia, Jundiaí, BRA; 3 Diagnósticos da América S.A., Hospital 9 de Julho, São Paulo, BRA; 4 Radiology, Hospital das Clínicas Faculdade de Medicina, Universidade de São Paulo, São Paulo, BRA; 5 Ortopedia e Traumatologia, Hospital do Coração - Hcor, São Paulo, BRA

**Keywords:** adhesive capsulitis, frozen shoulder, magnetic resonance imaging, radiological-clinical correlation, range of motion

## Abstract

Introduction

Adhesive capsulitis, or "frozen shoulder," is characterized by pain and significant functional limitation. It predominantly affects individuals over 40 years old and is commonly associated with systemic conditions such as diabetes mellitus and thyroid disorders. Imaging studies, particularly magnetic resonance imaging (MRI), may help understand structural changes linked to mobility restrictions.

Methods

This retrospective study analyzed patients clinically diagnosed with adhesive capsulitis between 2017 and 2019. All patients underwent non-contrast MRI on a 1.5T scanner. Radiological findings correlated with shoulder range-of-motion deficits, including capsular thickening in the axillary recess and hypersignal in multiple quadrants.

Results

Capsular thickening in the axillary recess showed a significant negative correlation with elevation (r = -0.42; p = 0.003) and lateral rotation (r = -0.38; p = 0.008). However, hypersignal in multiple quadrants did not correlate statistically with functional limitations.

Conclusion

Non-contrast MRI effectively identified capsular changes associated with mobility restrictions in adhesive capsulitis, aiding in diagnosis and management. Further studies with larger samples and longitudinal follow-ups are needed to validate these findings.

## Introduction

Adhesive capsulitis, or frozen shoulder, is characterized by pain and limited shoulder movement [1]. It accounts for approximately 13% of specialist consultations and is more prevalent in women over 40 [2]. Its occurrence is associated with comorbidities such as diabetes mellitus, thyroid disorders, and ethnic factors [3,4]. The pathophysiology of adhesive capsulitis involves thickening and contraction of the joint capsule and synovium, leading to progressive fibrosis and significantly impacting patients' quality of life [5].

The diagnosis is primarily clinical, but magnetic resonance imaging (MRI) has emerged as a valuable additional tool, allowing the identification of specific features such as capsular thickening in the axillary recess, pericapsular edema, and obliteration of fat in the rotator interval [6-8]. Previous studies suggest that these MRI findings may offer greater diagnostic accuracy and assist in differentiating it from other conditions [7]. Furthermore, Chellathurai et al. [9] proposed an MRI-based staging system that correlates imaging parameters, such as thickening of the inferior glenohumeral ligament and fat obliteration in the subcoracoid triangle, with clinical stages of the disease, reinforcing the utility of this approach for a more detailed and targeted diagnosis.

Despite the growing use of MRI to evaluate adhesive capsulitis, literature still lacks consensus on which imaging findings are most relevant for diagnosis and how they correlate with the clinical stages of the disease [10]. The absence of uniform criteria limits its applicability in clinical management and therapeutic planning [11].

Our study aims to investigate the correlation between non-contrast MRI findings and clinical variables in patients with adhesive capsulitis.

## Materials and methods

Study design

This cross-sectional study evaluated patients clinically diagnosed with adhesive capsulitis of the shoulder at a single institution between 2017 and 2019. The study was approved by the institution's Research Ethics Committee, and all participants signed an informed consent form before their inclusion.

Eligibility criteria

Adult patients diagnosed with adhesive capsulitis were included, defined according to Itoi et al.’s [12] criteria, including idiopathic stiff shoulder, global functional loss of active and passive movement, significant pain, and normal radiographs. All participants were over 18 years old, and the clinical diagnosis was confirmed by a shoulder specialist with over 18 years of experience. Patients were excluded if they had rotator cuff tears, glenohumeral osteoarthritis, calcific tendinopathy, or other structural causes of pain. Additionally, patients with prior surgery on the affected shoulder, infection, neoplasms, or neurological conditions that could impact the shoulder were not included.

Magnetic resonance imaging evaluation

All MRI scans were performed using a 1.5 T machine (HDxt, GE Medical Systems, Milwaukee, WI, USA) with a dedicated three-channel shoulder coil. Patients were positioned supine with their arms in a neutral position. No gadolinium, either intra-articular or intravenous, was used during the examinations. The protocol included the following sequences: axial, oblique coronal, and oblique sagittal intermediate-weighted images with fat suppression (repetition time [TR]: 2717-3784 ms; echo time [TE]: 42-46 ms; field of view [FOV]: 15 cm; slice thickness: 3-4 mm; matrix: 288 x 192); and oblique coronal and oblique sagittal T1-weighted images (TR: 350-517 ms; TE: minimum; FOV: 14-15 cm; slice thickness: 3-4 mm; matrix: 288 x 192). The images were blindly evaluated using the Osirix 9.0 software (Pixmeo SARL, Bernex, Switzerland) by a radiologist specializing in musculoskeletal imaging.

Analyzed variables

Range of Motion

A senior orthopedic researcher with 18 years of experience in shoulder and elbow conditions evaluated the range of motion.

Elevation (active shoulder flexion, measured in the sagittal plane), lateral, and medial rotation were assessed in the affected and contralateral healthy shoulders for comparison. Elevation and lateral rotation were measured in degrees using a manual goniometer, while medial rotation was evaluated based on the patient's hand position relative to the spinous processes. These values were converted into continuous numbers using a scale from 1 to 19 (T1 to T12 = 1 to 12, L1 to L5 = 13 to 17, sacrum = 18, and greater trochanter = 19), as described in a previous study [13].

Magnetic Resonance Imaging

All MRI variables were analyzed by a radiologist with over nine years of experience in musculoskeletal disorders. The radiologist was blinded to the patients' clinical findings.

Patients were categorized based on the presence or absence of high signal intensity in the axillary recess and joint capsule quadrants. This classification was performed using T2-weighted fat-suppressed images, with high signal intensity defined as areas of increased hyperintensity compared to surrounding soft tissues. For the evaluation of the joint capsule, patients were stratified into groups with and without high signal intensity in three or more quadrants.

The rotator interval infiltration was graded as mild/moderate (<50%) or severe (>50%) based on the extent of soft tissue hyperintensity relative to the rotator interval area on coronal T2-weighted images. This semi-quantitative assessment was performed by comparing the infiltrated area with the total rotator interval, using visual estimation supported by anatomical landmarks.

The capsule thickness in the axillary recess was measured in millimeters on the oblique coronal T2-weighted image with fat saturation, taken perpendicularly at the site of greatest thickening; similarly, the thickness of the coracohumeral ligament was measured in millimeters on the sagittal T1-weighted image at its thickest portion.

Statistical analysis

The statistical analyses were performed using SPSS software version 26.0 (IBM Corp., Armonk, NY, USA). Quantitative data were presented as means and standard deviations, while qualitative data were reported as absolute frequencies and percentages. The normality of continuous variables was assessed using the Shapiro-Wilk test to determine the appropriate statistical tests for comparisons.

For intergroup comparisons of non-normally distributed data, the Mann-Whitney U test was applied for two-group comparisons, and the Kruskal-Wallis test was used for comparisons involving more than two groups. When the Kruskal-Wallis test indicated significant differences, post-hoc pairwise comparisons with Bonferroni correction were conducted to adjust for multiple testing.

Correlations between radiological and clinical variables were evaluated using Spearman’s rank correlation coefficient. Effect sizes were calculated where applicable to assess the strength of associations. A p-value of less than 0.05 was considered statistically significant.

The required sample size was calculated based on an expected strong correlation (r = 0.6) between capsular thickness and range of motion deficits, with an alpha level of 0.05 and a power of 80%. Considering a 10% potential loss, the minimum sample size required was estimated to be 48 participants.

## Results

The sample consisted of 50 patients with a mean age of 56.1 ± 7.7 years, of whom 56% (n = 28) were female. The right side was the most affected, accounting for 54% (n = 27) of cases. The mean disease duration was 5.0 ± 4.1 months. Among comorbidities, 16% (n = 8) had diabetes mellitus, 32% (n = 16) had hypertension, and 10% (n = 5) had hypothyroidism (Table 1).

**Table 1:** Patients' clinical baseline SD, standard deviation

Characteristic	Value
Age (mean ± SD, years)	56.1 ± 7.7
Female (%)	56%
Right side affected (%)	54%
Duration of symptoms (mean ± SD, months)	5.0 ± 4.1
Diabetes mellitus (%)	16%
Hypertension (%)	32%
Hypothyroidism (%)	10%

**Figure 1:**
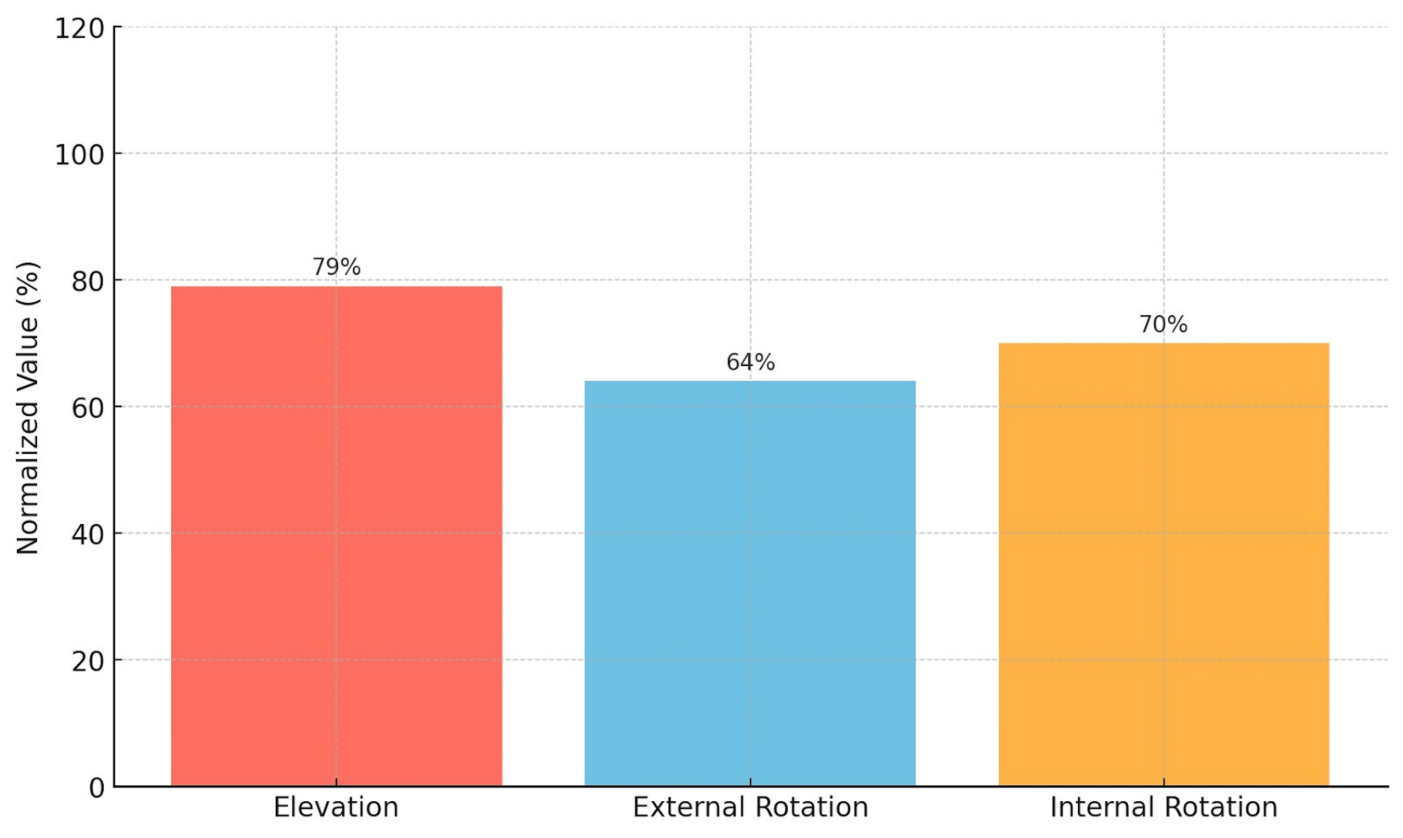
Percentage of the affected shoulder's range of motion compared to the normal shoulder.

In the mobility assessment, the elevation of the affected shoulder was 140 ± 22 degrees compared to 177 ± 8 degrees in the contralateral shoulder, resulting in a delta elevation of -37 ± 22 degrees (p < 0.001). The lateral rotation of the affected shoulder was 41 ± 18 degrees compared to 64 ± 11 degrees on the contralateral side, with a delta lateral rotation of -23 ± 19 degrees (p < 0.001). Medial rotation was measured with a mean of 15 ± 3 (approximately L3) on the affected side versus 10 ± 2.0 (approximately T10) on the contralateral side, resulting in a delta medial rotation of 4.6 ± 2.8 (p < 0.001), as shown in Table 2 and Figure 1.

**Table 2:** Range-of-motion ratio between affected and unaffected shoulders Data are presented as mean ± standard deviation. Statistical analysis was performed using the Mann-Whitney U test. Significance level was set at p < 0.05 SD, standard deviation

Variable	Affected, mean ± SD	Contralateral, mean ± SD	% to Contralateral	p-Value
Elevation (°)	140 ± 22	177 ± 8	79	<0.001
External rotation (°)	41 ± 18	64 ± 11	64	<0.001
Internal rotation	15 ± 3	10 ± 2	70	<0.001

**Figure 2:**
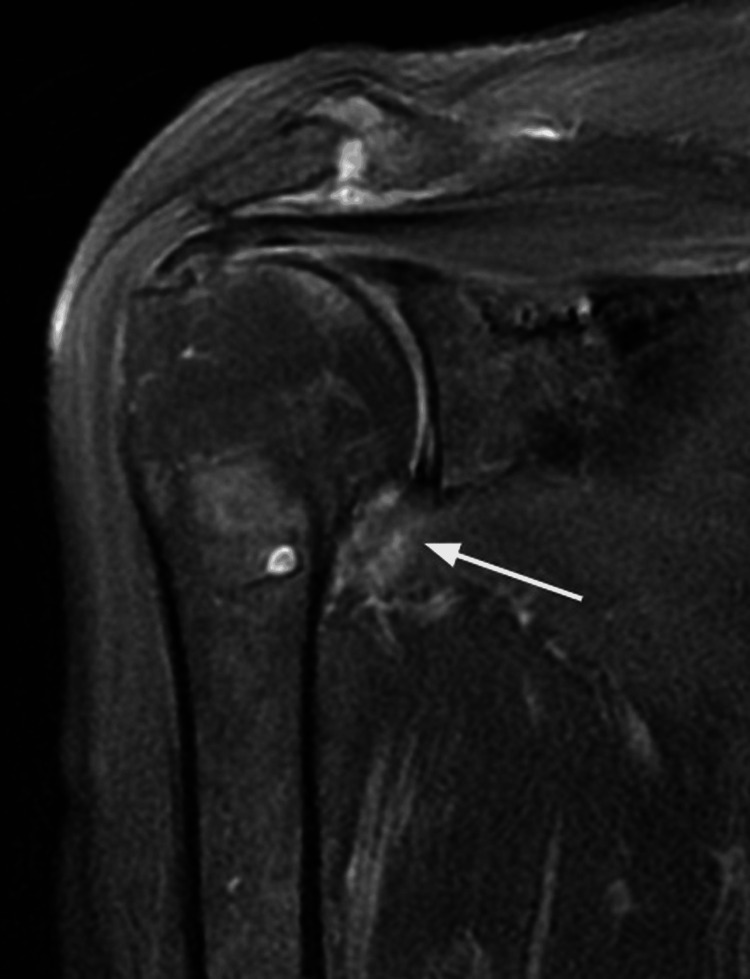
Coronal T2-weighted MRI. The white arrow indicates edema in the axillary recess.

**Figure 3:**
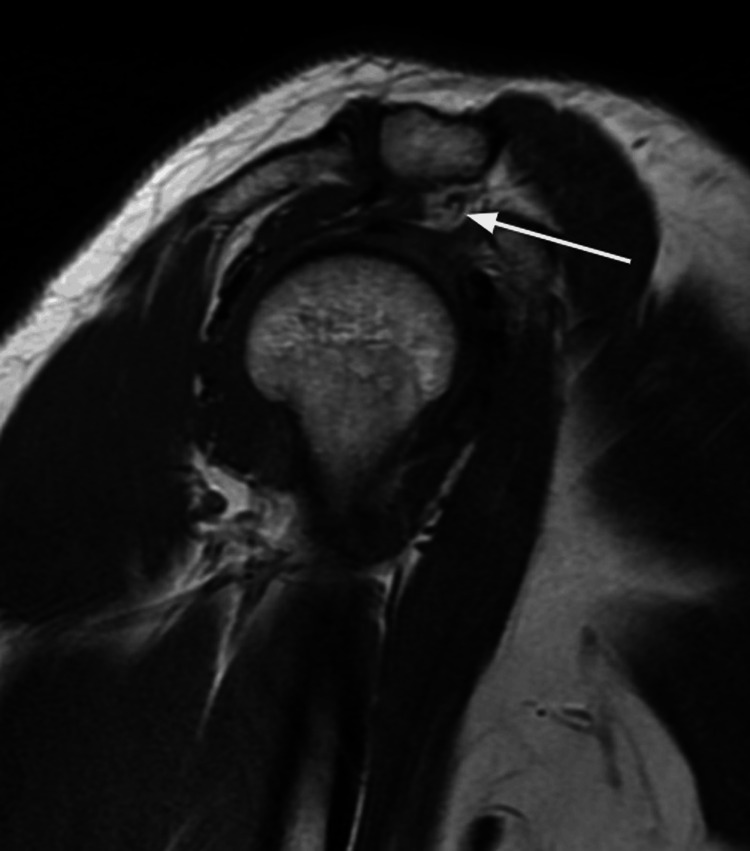
Sagittal T1-weighted MRI. The white arrow indicates obliteration of the rotator interval.

**Figure 4:**
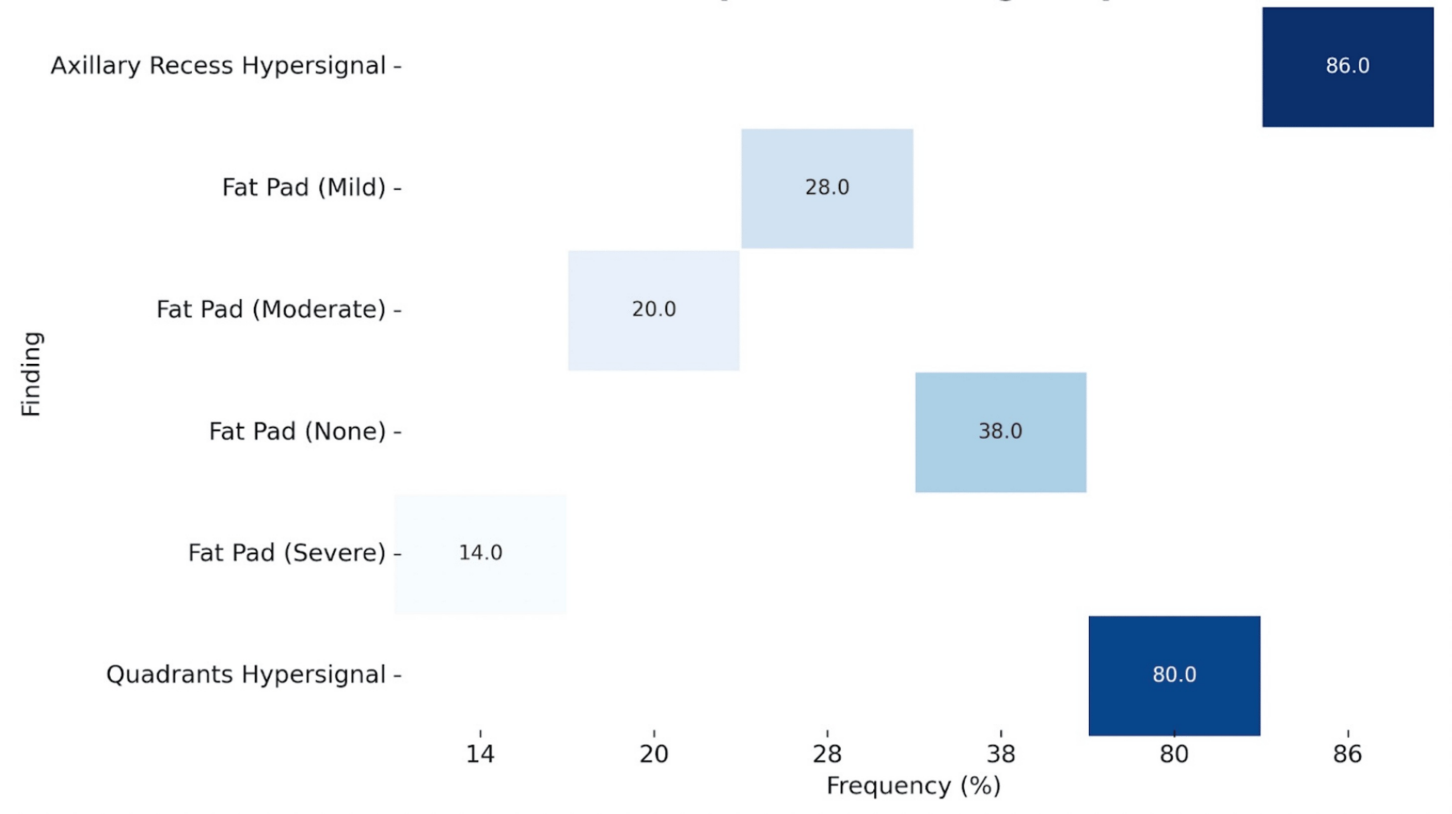
Heatmap of MRI finding frequencies.

Regarding the MRI findings, high signal intensity in the axillary recess (Figure 2) was identified in 86% (n = 43) of patients. In comparison, high signal intensity in three or more quadrants was present in 80% (n = 40). Obliteration of the rotator interval fat pad was absent in 38% (n = 19), mild in 28% (n = 14), moderate in 20% (n = 10), and severe in 14% (n = 7), as shown in Figure 3. These changes are shown in Figure 4.

Correlation between MRI findings and range of motion

Regarding high signal intensity in the axillary recess, the mean elevation deficit was -39 ± 23 degrees in the group with high signal intensity compared to -29 ± 16 degrees in the group without high signal intensity (p = 0.263). The lateral rotation deficit was -24 ± 20 degrees in patients with high signal intensity compared to -21 ± 7 degrees in those without this finding (p = 0.841). For medial rotation, the means were similar between the groups, with 4.5 ± 3 in the group with high signal intensity and 4.3 ± 1.9 in the group without high signal intensity (p = 0.912) (Table 3).

**Table 3:** Association between signal intensity in the axillary recess and range of motion Data are presented as mean ± standard deviation. Statistical analysis was performed using the Mann-Whitney U test. Significance level was set at p < 0.05.

Axillary recess	With hypersignal	Without hypersignal	p-Value
Elevation deficit (°)	-39 ± 23	-29 ± 16	0.263
External rotation deficit (°)	-24 ± 20	-21 ± 7	0.841
Internal rotation	4.5 ± 3.0	4.3 ± 1.9	0.912

When analyzing high signal intensity in three or more quadrants of the joint capsule, the elevation deficits were -37 ± 21 degrees in patients with this finding and -40 ± 25 degrees in those without (p = 0.689). Lateral rotation deficits had similar values between the groups, with -24 ± 19 degrees in the group with high signal intensity and -23 ± 18 degrees in the negative group (p = 0.881). For medial rotation, the mean values were also comparable, with 4.5 ± 2.8 degrees in the positive group and 4.6 ± 3.2 degrees in the negative group (p = 0.764). Data are presented in Table 4.

**Table 4:** Comparison of shoulder range of motion deficits in patients with and without high signal intensity in three or more joint capsule quadrants Data are presented as mean ± standard deviation. Statistical analysis was performed using the Mann-Whitney U test. Significance level was set at p < 0.05.

Three or more quadrants	Hypersignal	No hypersignal	p-Value
Elevation deficit (°)	-37 ± 21	-40 ± 25	0.689
External rotation deficit (°)	-24 ± 19	-23 ± 18	0.881
Internal rotation	4.5 ± 2.8	4.6 ± 3.2	0.764

Regarding rotator interval infiltration, the elevation deficit was -33 ± 19 degrees in negative cases, -36 ± 20 degrees in cases with mild/moderate infiltration (<50%), and -57 ± 27 degrees in cases with severe infiltration (>50%) (p = 0.0319). External rotation deficit was -21 ± 18 degrees in the group without infiltration, -24 ± 19 degrees in mild/moderate cases, and -30 ± 20 degrees in severe cases (p = 0.520). For medial rotation, the values were 4.1 ± 2.8 in the group without infiltration, 4.5 ± 2.9 in mild/moderate cases, and 5.6 ± 3.4 in severe cases, with no statistical significance (p = 0.489), as shown in Table 5.

**Table 5:** Association between rotator interval infiltration and range of motion Data are presented as mean ± standard deviation. Statistical analysis was performed using the Kruskal-Wallis test. Significance level was set at p < 0.05.

Rotator interval infiltration	No	Mild/moderate (<50%)	Severe (>50%)	p-Value
Elevation deficit (°)	-33 ± 19	-36 ± 20	-57 ± 27	0.032
External rotation deficit (°)	-21 ± 18	-24 ± 19	-30 ± 20	0.520
Internal rotation	4.1 ± 2.8	4.5 ± 2.9	5.6 ± 3.4	0.489

**Figure 5:**
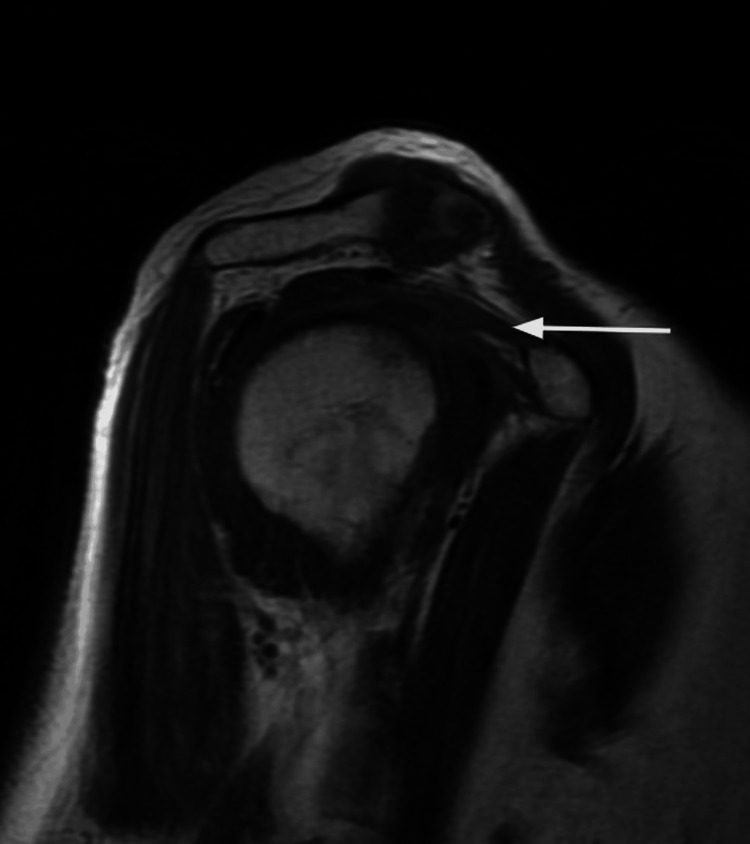
Sagittal T1-weighted MRI. The white arrow indicates thickening of the coracohumeral ligament.

Additionally, capsular thickness in the axillary recess showed significant negative correlations with elevation deficit (r = -0.42; p = 0.003) and external rotation deficit (r = -0.38; p = 0.008). In contrast, coracohumeral ligament thickness (Figure 5) did not show significant correlations with functional variables, with coefficients close to zero for elevation deficit (r = -0.02; p = 0.912), external rotation deficit (r = 0.05; p = 0.694), and internal rotation (r = -0.04; p = 0.815), indicating no relevant association (Table 6).

**Table 6:** Correlations between capsular thickness in the axillary recess, coracohumeral ligament thickness, and range of motion deficits Data are presented as correlation coefficients (r) and p-values. Statistical analysis was performed using Spearman’s rank correlation coefficient, followed by post-hoc pairwise comparisons with Bonferroni correction when applicable. Significance level was set at p < 0.05.

Variable	Capsular thickness in the axillary recess (r, p)	Coracohumeral ligament thickness (r, p)
Elevation deficit (°)	r = -0.42; p = 0.003	r = -0.02; p = 0.912
External rotation deficit (°)	r = -0.38; p = 0.008	r = 0.05; p = 0.694
Internal rotation	No correlation	r = -0.04; p = 0.815

## Discussion

This study demonstrated a significant association between the radiological variables of capsule thickness in the axillary recess and rotator interval infiltration with mobility limitations in shoulders affected by adhesive capsulitis. These results align with those reported by Zhao et al. [4], who identified similar correlations between capsule thickness and range of motion. However, discrepancies were noted regarding the findings of rotator interval infiltration, which are not consistently emphasized in comparable studies [6].

Capsule thickness in the axillary recess was an essential marker in our study, showing a negative correlation with shoulder elevation and lateral rotation. This finding aligns with the results reported by Dupont et al. [7], who identified capsule thickness as a relevant criterion for staging adhesive capsulitis. Additionally, the values observed in our cohort (mean of 4.2 mm) are consistent with those described in a recent study [10]. Similarly, Choi and Kim [13] noted that maximum axillary capsular thickening negatively correlates with a range of motion, reinforcing the importance of capsule thickness as an indicator of clinical severity in the early stages of adhesive capsulitis. Interestingly, Park et al. [14] observed that capsule thickness in the glenoidal portion of the axillary recess does not significantly correlate with functional limitations, which may explain differences among studies due to variations in measurement techniques and evaluated populations.

Although high signal intensity in three or more quadrants was identified in a large percentage of patients, our study did not find a statistically significant association between this finding and mobility limitations. This result aligns with Park et al. [14], who also reported variability in capsular edema findings and their correlations with functional deficits. On the other hand, Chellathurai et al. [9] emphasized the importance of pericapsular edema in clinical staging, particularly in the early stages of adhesive capsulitis, suggesting that the relevance of these findings may vary depending on the disease stage.

Furthermore, Choi and Kim [13] did not observe significant differences in the range of motion associated with edema in multiple quadrants, highlighting the need for caution when interpreting this parameter as a marker of severity. Ahn et al. [15] suggested that periarticular edema might be more closely related to pain than to functional limitations, which could explain the lack of direct correlation with mobility deficits. These findings underscore the need for standardized imaging criteria and further studies to explore better the role of high signal intensity in different stages of adhesive capsulitis.

Moreover, coracohumeral ligament thickness did not significantly correlate with clinical variables in our study. This result is consistent with the findings of Choi and Kim [13], who also did not identify a significant relationship between coracohumeral ligament thickness and functional mobility limitations, suggesting that its thickness may not be a reliable marker of clinical severity in adhesive capsulitis. Park et al. [14] reinforced this lack of correlation when studying inflammatory parameters of the ligament in different stages of the disease.

However, Pessis et al. [16] reported a potential association between the coracohumeral ligament and early inflammatory stages of adhesive capsulitis, emphasizing that ligament thickening may be more evident in the initial phases of the disease when the inflammatory process predominates. Additionally, Dupont et al. [7] suggested that ligament thickening might play a limited but relevant role in identifying the early stages of the disease, mainly when assessed alongside other radiological markers, such as pericapsular edema.

The presence of high signal intensity in the axillary recess was identified in 86% of patients in our study; however, this finding did not show statistical significance in its correlation with mobility limitations. This result contrasts with the findings of Sofka et al. [6], who suggested that inflammatory edema in the axillary recess is associated with active synovitis and may be an early indicator of inflammatory phases of adhesive capsulitis and changes in range of motion. Similarly, Chellathurai et al. [9] proposed that inflammatory changes visible on MRI, including high signal intensity in the axillary recess, could be helpful for the clinical staging of the disease. On the other hand, Park et al. [14] and Choi and Kim [13] did not find a significant association between high signal intensity in the axillary recess and functional deficits, emphasizing that variability in imaging evaluation criteria and population characteristics may influence the results. These discrepancies in the literature highlight the need for further studies with greater methodological standardization to define the clinical role of high signal intensity in the axillary recess as a diagnostic and prognostic marker in adhesive capsulitis.

Our study's strength lies in the combined evaluation of clinical and radiological variables, enabling a more comprehensive approach to understanding adhesive capsulitis. Additionally, the standardization of assessment techniques minimized biases and enhanced the reproducibility of the results.

Our study has some limitations that should be considered. First, the small sample size may have led to type II errors. Future studies with larger cohorts are needed to confirm these findings. The retrospective design may have prompted biases, such as the lack of prospective control over clinical and imaging evaluations. The absence of longitudinal data prevents an analysis of the natural progression of the disease and the response to treatment over time.

Another factor that could be considered a limitation was the use of non-contrast MRI. However, comparative studies between MRI with and without contrast have not demonstrated significant differences between the methods. A current trend recommends non-contrast imaging to evaluate adhesive capsulitis [17]. Finally, there was no histopathological or arthroscopic analysis to correlate to the radiological findings, which could enhance diagnostic accuracy and validate the measurements performed.

## Conclusions

The findings of this study demonstrated an association between capsule thickness in the axillary recess and rotator interval infiltration with limitations in elevation and lateral rotation in patients with adhesive capsulitis.
